# The impact of metformin on survival in diabetic endometrial cancer patients: a retrospective population-based analysis

**DOI:** 10.1007/s40200-023-01358-3

**Published:** 2023-12-21

**Authors:** Mingaile Drevinskaite, Auguste Kaceniene, Donata Linkeviciute-Ulinskiene, Giedre Smailyte

**Affiliations:** 1 Laboratory of Cancer Epidemiology, National Cancer Institute, P. Baublio 3B, Vilnius Lithuania, LT-08406 Vilnius, Lithuania; 2 The Medical Clinic, Västerås Central Hospital, Västerås, Sweden; 3 Department of Public Health, Institute of Health Sciences, Faculty of Medicine, Vilnius University, Vilnius, Lithuania

**Keywords:** Endometrial cancer, Type 2 Diabetes Mellitus, Metformin, Overall survival, Cancer-specific survival

## Abstract

**Purpose:**

The aim of our study was to assess overall survival and cancer-specific survival in endometrial cancer patients with type 2 diabetes mellitus (T2DM) using metformin.

**Methods:**

Patients with endometrial cancer and T2DM during 2000–2012 period were identified from the Lithuanian Cancer Registry and the National Health Insurance Fund database. Cancer-specific and overall survival were primary outcomes.

**Results:**

In our study we included 6287 women with endometrial cancer out of whom 664 were diagnosed with T2DM (598 metformin users and 66 never users). During follow-up (mean follow-up time was 8.97 years), no differences in risk of endometrial cancer specific mortality was observed in diabetic patients treated with metformin (Hazard Ratio (HR) 0.87, 95% Confidence Interval (CI) 0.70–1.07). Overall mortality in the diabetic metformin ever users’ group was significantly higher compared with the non-diabetic endometrial cancer women (HR 1.17, 95% CI 1.03–1.32) and in the group of metformin never users with T2DM (HR 1.42, 95% CI 1.07–1.87).

**Conclusion:**

Our study results suggest no beneficial impact on overall and cancer-specific survival in endometrial cancer patients who were treated with metformin as part of their diabetes treatment.

**Supplementary Information:**

The online version contains supplementary material available at 10.1007/s40200-023-01358-3.

## Introduction

Endometrial cancer is the third most common female gynecologic malignant neoplasia after breast and cervical cancers, and in the fifth place among all female cancers worldwide [1]. According to the GLOBOCAN cancer statistics, there were 382,068 new endometrial cancer cases and 89,929 deaths worldwide in 2018 [2]. The age-adjusted incidence of endometrial cancer is higher in developed countries and it has continued to increase since 2000 in parallel with changing lifestyles and rising numbers of metabolic syndrome, obesity and type 2 diabetes mellitus (T2DM) [3, 4]. For instance, the highest incidence rates are observed in Northern and Western Europe [5]. In contrast, decreasing trends of endometrial cancer mortality are observed in most countries worldwide with the greatest decline recorded in developed countries [6].

Various studies have shown that the risk of endometrial cancer increases with early menstruation, family history of endometrial cancer, and long-term use of external estrogens for hormone therapy without progestin support [7]. Other well-established risk factors of endometrial cancer are obesity, T2DM, hyperinsulinemia, and insulin resistance [8, 9]. Endometrial cancer is a hormone dependent cancer and the peak of incidence is in postmenopausal women between 50 and 70 years old [10]. Obesity may have an impact on developing endometrial cancer due to fatty cells producing large amounts of estrogen, which levels are found to be increased in endometrial cancer patients [9]. T2DM is an independent risk factor for endometrial cancer and multiple studies have shown a positive association between diabetes and endometrial cancer [11, 12]. It is observed that women who develop endometrial cancer with comorbidities such as diabetes and obesity have a decreased life expectancy when compared with non-diabetic and non-obese women [13].

Traditionally, endometrial cancer is classified as type 1 which is more common and has better prognosis than poorly differentiated type 2 endometrial cancer [14]. In most cases, endometrial cancer is diagnosed in early stages; however, despite advances in the treatment, the prognosis of advanced stages remains poor [15]. Some epidemiologic studies have suggested the linkage between metformin use and reduced risk of developing endometrial cancer [16]. However, results are controversial and our knowledge are still limited.

Metformin is the first-line treatment for T2DM and it is known to increase insulin sensitivity, inhibit liver gluconeogenesis, and reduce hyperglycaemia [17]. In addition, metformin has demonstrated some direct and indirect molecular mechanisms in endometrial cancer cells as well as in some other cancers [18]. In vitro studies have shown that metformin induces apoptosis of endometrial cancer cells and inhibits cell proliferation in both normal and cancerogenous cells [19, 20].

To our knowledge, we performed the largest population-based retrospective cohort study that aimed to analyse the association of metformin and cancer-specific and overall survival in endometrial cancer patients and to contribute to the existing data that metformin use might reduce the risk of developing endometrial cancer.

## Materials and methods

This study was conducted according to the guidelines of the Declaration of Helsinki and approved by the Vilnius regional biomedical research ethics committee (approval number No. 158200-17-913-423 on 9 May 2017).

In total, 7115 primary endometrial cancer (International Classification of Diseases (ICD)-10 code C54) cases diagnosed between 2000 and 2012 were extracted from the Lithuanian Cancer Registry database, a nationwide and population-based cancer registry, which covers the whole territory of Lithuania and collects demographic, personal identification, and medical records about all new cancer cases. Available data for this analysis included date of birth, personal identification number, date of diagnosis and date of death, underlying cause of death, cancer site, histology and extent of disease. Stages were fitted to the current International Federation of Gynaecology and Obstetrics (FIGO) stage [21]. Endometrial cancers were categorized as endometrioid and non-endometrioid cancers according to their histology type. Information on diagnosis of T2DM (ICD-10 code E11), and antidiabetic medication was obtained from the National Health Insurance Fund (NHIF) database. To minimize the risk of false type 2 diabetes classification, T2DM status was assigned to patients who were reported as T2DM patients and received prescriptions of antidiabetic medications in the NHIF database. Data linkage between different databases was based on the personal identification code, which is unique to each resident of Lithuania. Only women with an estimated duration of T2DM of 1 full year before endometrial cancer diagnosis were included in the analyses.

**Fig. 1:**
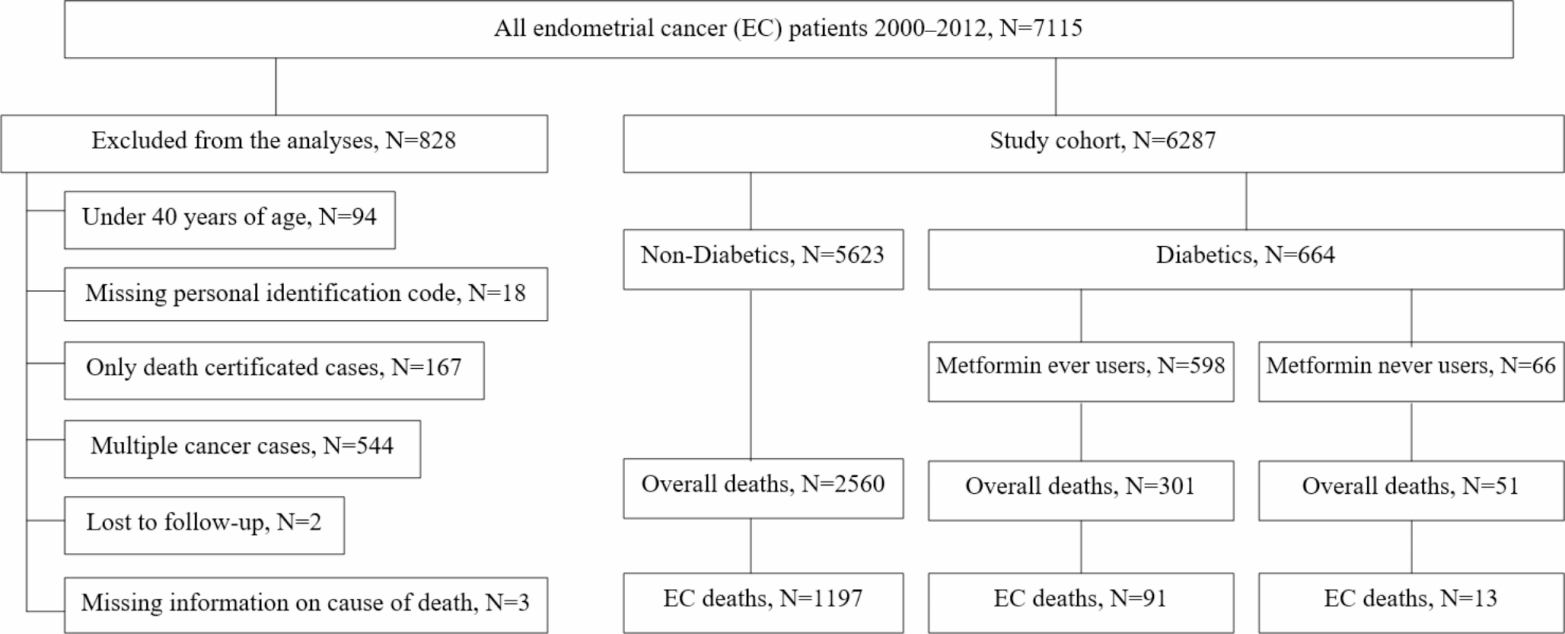
Flowchart of the study population

The final study cohort of 6287 women with endometrial cancer was formed after exclusion of patients with missing data, younger women than 40 years old, only death certificated cases and multiple cancer cases (Fig. 1).

There were 664 women with T2DM diagnosis who used antidiabetic medication for 365 at least days. To examine the exposure to metformin, patients with T2DM were divided into two groups depending on antidiabetic medication: “metformin ever users” (598 women who used metformin alone or in combination with other antidiabetic medication) and “metformin never users” (66 women who used other antidiabetic medication without metformin: sulfonylureas, thiazolidinediones, meglitidines, insulin). The follow-up started from the date of endometrial cancer diagnosis to the first of the following events: death, emigration, or the end of the follow–up (31 December 2019). Almost half of the cohort members (2912 women, 46%) had died by the end of the follow–up, and 1301 of them due to endometrial cancer.

Univariate and multivariate Cox Proportional Hazard regression models were performed to assess risk factors for overall and cancer-specific survival. Results were presented as hazard ratios (HR) with 95% confidence intervals (CI) and p value of < 0.05 were considered statistically significant. Multivariate models were adjusted for the following prognostic factors: age at endometrial cancer diagnosis, extent of disease, histological subtype, and metformin use. All statistical analyses were carried out using STATA, version 11; StataCorp., College Station, Texas, USA.

## Results

Demographic and clinico-pathological patient characteristics according to T2DM and metformin treatment are presented in Table 1. The mean age at diagnosis was higher in women with diabetes. The majority of women who never used metformin (71.2%) were aged 70 or more at endometrial cancer diagnosis. More women with diabetes had endometrioid histological subtype of endometrial cancer (67.2% of metformin ever users and 59.1% of metformin never users, respectively) compared with the non-diabetic women (50.9%). The majority of cases (64.8%) of endometrial cancer were with stage I at the time of diagnosis. The mean follow-up time was 8.97 years (range 0.003–19.97) and the median follow-up time was 9.08 years.

Overall mortality in the endometrial cancer metformin ever users‘ group was significantly higher compared with the non-diabetic endometrial cancer women (HR 1.17, 95% CI 1.03–1.32) after adjusting for all known prognostic factors. In addition, greater risk of death was in the group of metformin never users with T2DM (HR 1.42, 95% CI 1.07–1.87) (Table 2).

**Table 1:** Demographic and clinico-pathological patient characteristics according to T2DM and metformin treatment

Patient characteristics	Non-Diabetics	Metformin ever users	Metformin never users	Total
Number of patients	N = 5623 (89.4%)	N = 598 (9.5%)	N = 66 (1.1%)	N = 6287 (100%)
Mean age at diagnosis. years (+/− SD*)	63.8 (10.8)	66.4 (8.2)	72.7 (7.9)	64.1 (10.6)
Age at diagnosis, years				
40–50	542 (9.6)	12 (2.0)	1 (1.5)	555 (8.8)
50–59	1534 (27.3)	107 (17.9)	4 (6.1)	1645 (26.2)
60–69	1793 (31.9)	268 (44.8)	14 (21.2)	2075 (33.0)
≥ 70	1754 (31.2)	211 (35.3)	47 (71.2)	2012 (32.0)
Histology				
Endometrioid	2864 (50.9)	402 (67.2)	39 (59.1)	3305 (52.6)
Non-endometrioid	2472 (44.0)	178 (29.8)	24 (36.4)	2674 (42.5)
Unknown	287 (5.1)	18 (3.0)	3 (4.5)	308 (4.9)
Stage				
I	3604 (64.0)	432 (72.2)	41 (62.1)	4077 (64.9)
II	749 (13.3)	66 (11.0)	8 (12.1)	823 (13.0)
III	560 (10.0)	31 (5.2)	6 (9.1)	597 (9.5)
IV	285 (5.1)	18 (3.1)	3 (4.6)	306 (4.9)
Unknown	425 (7.6)	51 (8.5)	8 (12.1)	484 (7.7)

*SD – Standard Deviation

**Table 2:** Univariate and multivariate Cox Proportional Hazard regression model estimates for overall mortality by demographic and clinico-pathological patient characteristics

	Univariate				Multivariate			
Variable	HR*	95% CI**		p	HR	95% CI		p
Age at diagnosis, years								
40–50	1.00				1.00			
50–59	1.96	1.54	2.50	< 0.001	2.03	1.60	2.58	< 0.001
60–69	3.75	2.98	4.72	< 0.001	3.90	3.10	4.91	< 0.001
≥ 70	9.46	7.54	11.87	< 0.001	9.39	7.47	11.79	< 0.001
Diabetes								
No	1.00				1.00			
Yes	1.28	1.14	1.43	< 0.001	1.20	1.07	1.34	0.002
Antihyperglycemic medication								
Non-Diabetics	1.00				1.00			
Metformin ever users	1.19	1.06	1.34	0.004	1.17	1.03	1.32	0.013
Metformin never users	2.33	1.76	3.07	< 0.001	1.42	1.07	1.87	0.014
Histology								
Endometrioid	1.00				1.00			
Non-endometrioid	1.52	1.41	1.64	< 0.001	1.42	1.31	1.53	< 0.001
Unknown	2.96	2.55	3.42	< 0.001	2.36	2.02	2.75	< 0.001
Stage								
I	1.00				1.00			
II	1.62	1.45	1.79	< 0.001	1.46	1.31	1.62	< 0.001
III	3.03	2.72	3.37	< 0.001	3.04	2.73	3.39	< 0.001
IV	12.36	10.85	14.05	< 0.001	10.11	8.86	11.54	< 0.001
Unknown	1.64	1.43	1.89	< 0.001	1.59	1.38	1.84	< 0.001

*HR – Hazard Ratio

**CI – Confidence Interval

Reverse associations with diabetes and metformin use was observed in endometrial cancer specific survival analysis (Table 3). Compared with non-diabetic cancer patients, lower risk of endometrial cancer specific mortality was observed in patients with T2DM treated with metformin in univariate (HR 0.71, 95% CI 0.58–0.88) analysis, although after adjustment outcome did not reach statistical significance. In the Cox regression analysis, older age, non-endometrioid histology and more advanced cancer stage were associated with an increase in both overall and in endometrial cancer specific mortality.

**Table 3:** Univariate and multivariate Cox Proportional Hazard regression (HR) model estimates for endometrial cancer specific mortality by demographic and clinico-pathological patient characteristics

	Univariate				Multivariate			
Variable	HR	95% CI		p	HR	95% CI		p
Age at diagnosis, years								
40–50	1.00				1.00			
50–59	1.55	1.15	2.10	0.004	1.66	1.23	2.25	0.001
60–69	2.36	1.77	3.15	< 0.001	2.52	1.89	3.37	< 0.001
≥ 70	4.47	3.37	5.93	< 0.001	4.35	3.27	5.78	< 0.001
Diabetes								
No	1.00				1.00			
Yes	0.74	0.61	0.91	0.004	0.85	0.70	1.04	0.122
Antihyperglycemic medication								
Non-Diabetics	1.00				1.00			
Metformin ever users	0.71	0.58	0.88	0.002	0.87	0.70	1.07	0.190
Metformin never users	1.07	0.62	1.85	0.800	0.77	0.44	1.33	0.347
Histology								
Endometrioid	1.00				1.00			
Non-endometrioid	1.97	1.75	2.21	< 0.001	1.67	1.44	1.83	< 0.001
Unknown	4.89	4.04	5.93	< 0.001	3.17	2.59	3.88	< 0.001
Stage								
I	1.00				1.00			
II	2.46	2.08	2.91	< 0.001	2.15	1.82	2.54	< 0.001
III	6.69	5.78	7.75	< 0.001	6.37	5.49	7.38	< 0.001
IV	24.98	21.27	29.34	< 0.001	19.81	16.77	23.40	< 0.001
Unknown	2.46	1.99	3.04	< 0.001	2.18	1.75	2.71	< 0.001

CI – Confidence Interval

## Discussion

One Lithuanian study, as well as other epidemiologic studies, have shown a strong association between diabetes and the incidence of endometrial cancer [22, 23]. However, results from studies which have analysed metformin association with endometrial cancer patients‘ survival outcomes are still conflicting [11]. In our study, we observed that after adjusting for all known prognostic factors, overall mortality of endometrial cancer patients who used metformin was significantly higher compared with the non-diabetic endometrial cancer women. Reverse results were observed in the *Urpilainen et al.* study where they did not find any association between endometrial cancer patients treated with metformin and better overall survival (HR 0.86, 95% CI 0.41–1.79). However, as a limitation, it should be noted that the sample size was not very extensive due to the single-institution based records [24]. Another study, who analysed overall survival in diabetic endometrial cancer women using propensity score matching, observed that metformin users had similar overall survival outcomes compared with other diabetic women with endometrial cancer (HR 0.61, 95% CI 0.30–1.23) [25]. Though a recent meta-analysis reported that metformin could significantly improve the overall survival in metformin users versus non-users in endometrial cancer patients with T2DM (HR 0.57, 95% CI 0.42–0.78), no significant difference in overall survival was found between the patients with diabetes who used metformin and the women without diabetes [26]. In a retrospective cohort analysis by *Ko et al.*, it was observed that metformin use was associated with improved overall survival in diabetic endometrial cancer patients. In their study, diabetic endometrial cancer patients who did not use metformin were 2.3 (95% CI 1.3–4.2) times more likely to die of all causes compared with metformin users. However, they did not find any significant overall survival difference in patients with endometrioid and non-endometrioid endometrial cancer histologies [27]. In addition, another study showed the beneficial effect on overall survival using metformin only in the non-endometrioid endometrial cancer group, and the patients with endometrioid adenocarcinoma did not benefit from metformin use [28].

In our study, for cancer-specific mortality we found no differences in the metformin ever users‘ group compared with patients without diabetes. In the study by *Arima et al.* authors did not observe endometrial cancer related mortality differences in metformin users compared with patients who used other forms of antihyperglycemic medication. They hypothesized that the results could have been affected by age due to the fact that metformin users were on average seven years younger than the patients on other oral antidiabetic drugs [29]. In addition to *Arima et al.*, similar results were observed in a cohort study by *Seebacher et al.* who analysed overall and cancer-specific survival in the total cohort and in a subgroup of overweight patients. They did not find any association between metformin users (both overweight and in the total cohort) compared with other diabetes drugs and better cancer-specific survival. However, they demonstrated a significant difference between overall survival. Over-weight diabetic patients who had never used metformin had a HR of 2.3 (95% CI 1.1–4.7) chance of dying from any cause compared with patients without diabetes and diabetic patients using metformin. The authors noted that after adjusting to other known prognostic factors and stage, the effect of metformin did not remain significant for overall survival [30]. By contrast, *Feng J-L et al.* reported that metformin was associated with improved cancer-specific survival in women with endometrial cancer (HR 0.95, 95% CI 0.90–0.99) [31].

Our population-based cohort study has several strengths. First, we have studied to the best of our knowledge the largest cohort comprising a total of 6287 women with endometrial cancer diagnosis out of whom 664 women had diagnosis of T2DM and 598 were metformin users. The second important strength is the long follow-up of more than 8 years.

The limitations of our study are the following. Firstly, it is a retrospective study. Secondly, the lack of data on important risk factors for mortality, including body mass index, other used drugs, and other comorbidities. Thirdly, users of metformin and never users were compared with a non-diabetic population. This makes it challenging to analyse results because both groups are heterogeneous. In the metformin ever users group, metformin is usually used as first-line therapy, and it might be used in combination with other diabetes drugs, whereas never users comprised patients who have never used metformin in any combination of anti-diabetic medications. In addition, the vast majority of women who never used metformin were aged 70 or more at the date of endometrial cancer diagnosis, and overall survival results could have been affected by comorbidities.

In conclusion, the results of our retrospective study suggest no beneficial effect of metformin on overall and cancer-specific survival. Overall mortality in the endometrial cancer metformin ever users‘ group was significantly higher compared with the non-diabetic endometrial cancer women and there were no differences in cancer-specific survival between non-diabetic endometrial cancer patients and diabetic metformin ever users and never users groups.

### Electronic supplementary material

Below is the link to the electronic supplementary material.

Supplementary Material 1
